# An optimised method for the production of MERS-CoV spike expressing viral pseudotypes

**DOI:** 10.1016/j.mex.2015.09.003

**Published:** 2015-10-13

**Authors:** K. Grehan, F. Ferrara, N. Temperton

**Affiliations:** Viral Pseudotype Unit, Medway School of Pharmacy, University of Kent, Chatham Maritime, Kent, United Kingdom

**Keywords:** MERS coronavirus, Lentiviral pseudotype, Virus neutralisation

## Abstract

The production and use of pseudotyped viral particles are widely established for many viruses, and applications in the fields of serology and vaccine development are manifold. Viral pseudotypes have proven to be powerful tools to study the effects of viral evolution on serological outcomes, viral tropism and immunogenicity studies. Pseudotyped viruses are chimeric constructs in which the outer (surface) glycoprotein(s) of one virus is combined with the replication-defective viral “core” of another virus. Pseudotypes allow for accurate, sequence-directed, sensitive antibody neutralisation assays and antiviral screening to be conducted within a low biosecurity facility and offer a safe and efficient alternative to wildtype virus use.

The protocol outlined here represents a rapid and reliable method for the generation of high-titre pseudotype viral particles with the MERS-CoV spike protein on a lentiviral core, and is adapted from previously published protocols. This protocol is optimised for transfection in a 100 mm Petri dish with 7 ml of supernatant harvested, however it can be readily scaled to different production volumes.

This protocol has a number of advantages including:

• Use of readily available reagents.
• Consistent, high virus titres.
• Rapid generation of novel glycoproteins for research into strain variation.

## Graphical abstract

**Figure:**
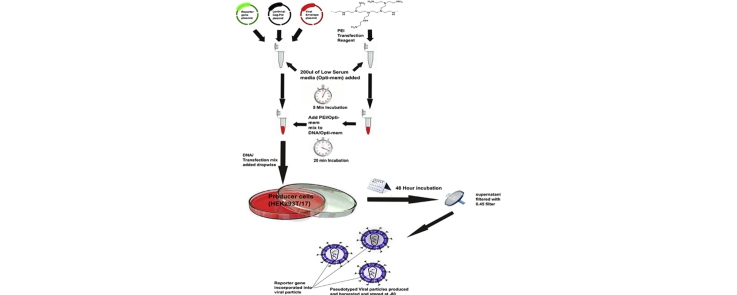

## Method details

### Materials and equipment

• HEK 293T/17 cells (ATCC^®^ CRL-11268™).
• Dulbecco's modified Eagle medium with Glutamax (Cat. No. 31966-021) supplemented with 10% foetal bovine serum and 1% penicillin/streptomycin (P/S).
• Trypsin–EDTA (0.05%), phenol red (Cat. No. 25300-054).
• Gibco reduced serum media Opti-MEM^®^ (Cat. No. 31985-047).
• Optional: TC20™ Automated Cell Counter (Cat. No. 145-0102EDU).
• Branched polyethyleneimine solution at concentration of 1 mg/ml (Cat. No. 408727).
• Sterile syringes (10 ml).
• Millex-HA 0.45 μm filters (Cat. No. SLHAM33SS).
• Rabbit polyclonal antibody to novel coronavirus (HCoV-EMC/2012) spike protein (SinoBiological Cat. No. 40069-RP02).
• Nunc^®^ UpCell™ surface cell culture dish (Manufacturer No. 174902).
• Microcentrifuge tube Safe-Lock write-on graduated with lid latch 1.5 ml.

*Note*: All steps should be carried out in a class II biosafety cabinet to avoid contamination.

### Plasmids

Glycoprotein expression plasmid: pCAGGS-MERS-CoV spike. (*Note*: The MERS-CoV spike protein should be codon optimised) [3], [5].

Lentiviral vector plasmid expressing firefly luciferase: pCSFLW [8], [11].

Second-generation lentiviral packaging construct plasmid: p8.91 (expressing HIV-gag) [8], [12].

### Transfection steps

**Timeline: Transfection – 24** **h**
  1. 293T/17 cells should be subcultured into 100 mm Petri dishes at a ratio that will yield 70–90% confluence at the time of transfection. In our hands this protocol yields similar results regardless of Petri dish size when supernatant yield is equivalent.
**Timeline: Day of transfection**
  2. DMEM/10% FBS/1% P/S and Opti-MEM^®^ should be pre-warmed to 37 °C using a water bath or similar.
  3. Prepare and label two sterile 1.5 ml microcentrifuge tubes (tube 1 and tube 2) per transfection.
  4. Add the following plasmids (0.9:1:1.5 envelope:core:vector ratio) for transfection to tube 1:
    a. pCAGGS-MERS-CoV spike: 0.9 μg.
    b. p8.91-lentiviral vector: 1.0 μg.
    c. pCSFLW: 1.5 μg.
  5. Add 200 μl Opti-MEM^®^ to the plasmid DNA mix (tube 1).
  6. Add 200 μl Opti-MEM^®^ and 35 μl of 1 mg/ml PEI to tube 2.
  7. *Incubation step*. Mix both tubes by gently flicking and incubate for 5 min at room temperature (RT).
  8. After incubation, pipette the Opti-MEM^®^/PEI solution from tube 2 into the Opti-MEM^®^/DNA solution in tube 1.
  9. *Incubation step*. Gently flicking the tube to mix every 3–4 min, incubate the tube at RT for 20 min.
  10. While transfection mix is incubating, the culture media on the 293T/17 cells should be removed and 7 ml of fresh DMEM/10% FBS/1% P/S added. It is important at this point to add media slowly to one side of the dish to avoid detaching adherent cells.
  11. After 20 min incubation, pipette the DNA/Opti-MEM^®^/PEI solution onto the 293T/17 cells by adding dropwise over the complete area of the plate. Swirl the plates gently to ensure even dispersal.
  12. *Incubation step*. Incubate the plate at 37 °C, 5% CO_2_ overnight (o/n). In our hands incubation times of between 12 and 16 h result in equivalent final pseudotype production titres.
**Timeline: 12–16 h post transfection**
  13. Post o/n incubation the media on the cells should be changed and 7 ml fresh DMEM/10% FBS/1% P/S added. Add media slowly to one side of the plate to avoid cell detachment.
  14. Incubate the plates 37 °C 5% CO_2_ o/n for 32–36 h.
**Timeline: 44–52 h post transfection**
  15. Supernatant containing the viral pseudotype particles are harvested using a 10 ml sterile syringe and then filtered into falcon tubes via a syringe driven Millex HA-0.45 μm filter.
  16. Store all filtered supernatant at −80 °C. It is recommended that supernatant is stored as aliquots to avoid multiple freeze thaw cycles. *Note*: Supernatant may be stored at 4 °C for up to one week with no detectable loss of titre.
  17. *Optional step*: Additional media may be added to cells to allow a second harvest 18–24 h later by adding further DMEM/10% FBS/1% P/S. In this case extreme care must taken in initial pseudotype collection (step 15) to avoid damage to cell monolayer. We have observed that cells in poor health after first harvest yield significantly less viral particles upon second harvest.

*Note*: A control pseudotype virus may be created by following the steps outlined above but leaving out the pCAGGS-MERS-CoV spike construct. This produces particles that do not express the viral surface glycoprotein and therefore should be unable to transduce target cells (Δ-env control) [3].

### Titration steps (Fig. 1)

*Note*: Titration consists of transduction of reporter (in this case firefly luciferase) into target cells mediated by the viral glycoprotein expressed on the viral pseudotype. Controls for titrations are provided via the inclusion of “cell only” and “Δ-env” columns.

**Fig. 1:**
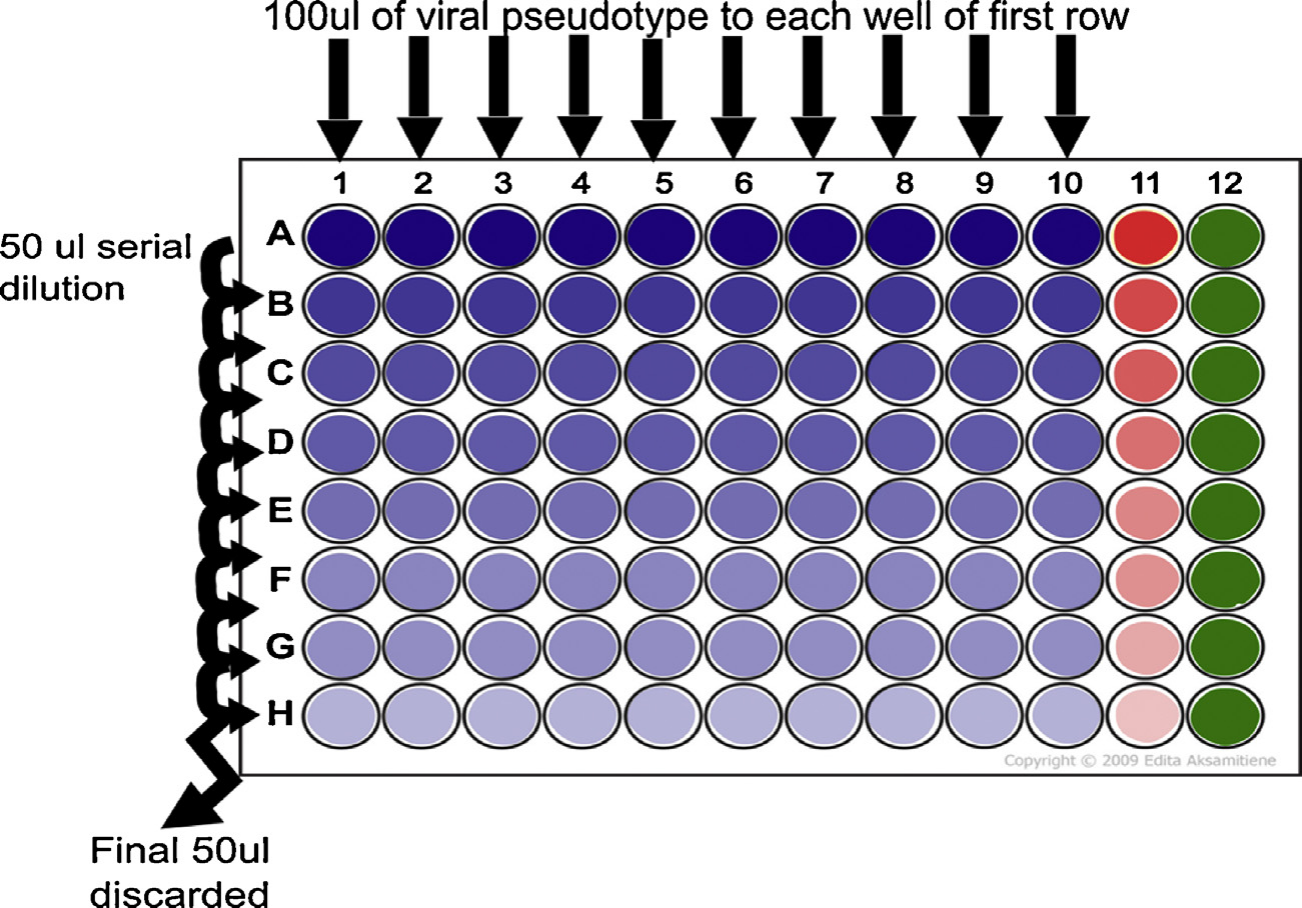
Serial dilution step showing addition of 100 μl of pseudotype virus supernatant to each well of row A and dilution of 50 μl taken from this well to row B. This process is continued to end of plate (row H) at which point the final 50 μl is discarded. Δ-Env control is indicated in red (column 11) and cell only control is indicated in green (column 12). (For interpretation of the references to colour in this figure legend, the reader is referred to the web version of this article.)

1. In a 96 well white plate add 50 μl of DMEM/10% FBS/1% P/S to the entire column of “cell only” control (see Fig. 1 column 12).
2. Add 50 μl of DMEM/10% FBS/1% P/S from row B to H that are to contain pseudotyped virus or Δ-env control.
3. Add 100 μl of MERS pseudotype virus supernatant to each well of row A (excluding control columns) and add 100 μl of Δ-env to column 11 (see Fig. 1).
4. Remove 50 μl from row 1 virus-containing wells and perform 1:2 serial dilutions down all wells below.
5. With each dilution step use pipette to mix 8 times up and down.
6. After completing serial dilution the final 50 μl from the last well of each column should be discarded. *Note*: at this point each well should contain 50 μl of mixed DMEM and viral supernatant.
7. Prepare a plate of susceptible target cells (Huh-7) (preferentially subcultured 1:4 48 h before):
  a. Remove media from plate.
  b. Wash the plate with 2 ml of trypsin and discard trypsin.
  c. Add additional 2 ml of trypsin to the plate to detach cells.
  d. After cells have detached add DMEM/10% FBS/1% P/S to the plate to quench trypsin activity.
  e. Count cells using TC20™ Automated Cell Counter or haemocytometer and add 1x10^4^ cells in a total volume of 50 μl to each well.
8. Centrifuge plate for 1 min at 500 rpm if there are droplets on the sides of the wells.
9. Incubate the plate for 48 h at 37 °C 5% CO_2_.
10. Read plate using Bright Glo™ luciferase assay system or equivalent.

### Method validation and transfection results

**Fig. 2:**
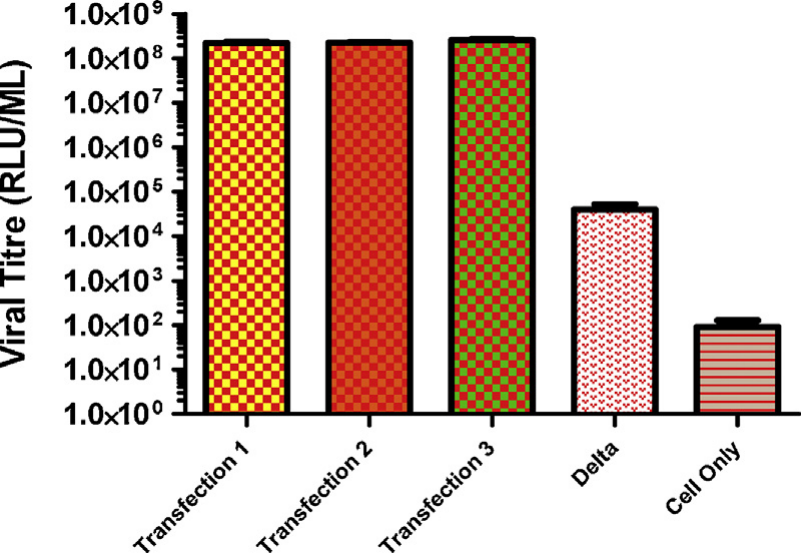
Pseudotype production titres from three replicates of optimised transfection protocol using codon optimised MERS-CoV Spike. Delta envelope titre overestimation in comparison to cell only control is related to the mathematical method that is used to calculate pseudotype titre and that cannot be applied to cell only.

Fig. 2 displays data recorded from multiple transfections indicating consistency of results. Results are measured in Relative Luminescence Units (RLUs) as measured using a GloMax^®^ 96 Microplate Luminometer and the Bright Glo™ luciferase assay system. The pseudotype particles generated in the absence of viral envelope (Delta) show increased luciferase activity compared to cell only in part due to transformation method used to discern RLU per ml. The presence of some carry-over luciferase within viral particles is also likely to generate an increase in RLU values recorded (Table 1).

**Fig. 3:**
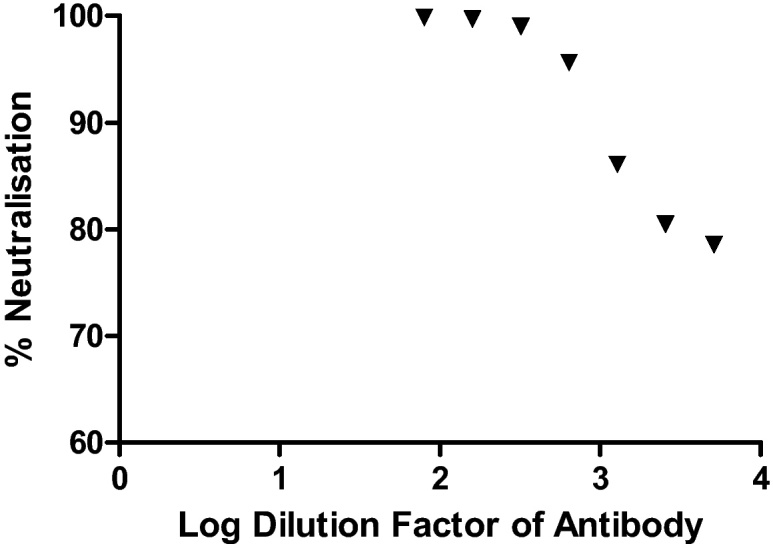
Anti-MERS-spike antibody (rabbit polyclonal antibody to novel coronavirus (HCoV-EMC/2012) spike protein) neutralises MERS viral pseudotype entry into Huh7 cells.

Fig. 3 shows percentage neutralisation of the MERS-CoV pseudotype with commercially produced anti-MERS spike antibody. Figure clearly indicates that as the dilution factor increases, so the percentage neutralisation decreases, 100% neutralisation indicates that RLU values at this concentration are equivalent to a delta envelope control.

The protocol outlined here provides a rapid and consistent method for the generation of high-titre viral pseudotype particles expressing the MERS-CoV spike protein suitable for further downstream applications [2], [4], [5], [7]. Efficient knock-down of pseudotype virus entry using a polyclonal antibody directed against the spike glycoprotein (Fig. 3) demonstrates potential utility for vaccine immunogenicity and Mab/antiviral screening [3]. The use of readily available reagents should facilitate increased reproducibility [1], [6], [9], [10], [11], [12].

## Figures and Tables

**Table 1:** Mean RLU calculated per ml of viral supernatant for three pseudotype production runs.

	Transfection 1	Transfection 2	Transfection 3	Cell only
Mean/ml titre	2.2E+08	2.3E+08	2.6E+08	91
